# Romanian Consensus Statement for Hormone Receptor-Positive and Human Epidermal Growth Factor Receptor 2-Negative Metastatic Breast Cancer (HR+/HER2– mBC) and Triple-Negative Metastatic Breast Cancer (mTNBC)

**DOI:** 10.3390/curroncol33020120

**Published:** 2026-02-17

**Authors:** Mircea Dragoș Median, Nicoleta Zenovia Antone, Simona Volovăț, Laura Mazilu, Șerban Mircea Negru, Răzvan Ovidiu Curcă, Amedeia Niță, Raluca Ileana Pătru, Andrei Ungureanu, Vlad Lupu, Cristina Marinela Oprean

**Affiliations:** 1 Filantropia Clinical Hospital, 011132 Bucharest, Romania; 2 Breast Tumors Center, Institute of Oncology “Prof. Dr. Ion Chiricuta”, 400015 Cluj-Napoca, Romania; 3 Regional Institute of Oncology, 700483 Iași, Romania; 4 Department of Oncology and Radiotherapy, University of Medicine and Pharmacy “Grigore T. Popa”, 700115 Iași, Romania; 5 Ovidius Clinical Hospital, 905900 Constanța, Romania; 6 Ovidius University, 900527 Constanța, Romania; 7 Department of Oncology, Faculty of Medicine, ‘Victor Babes’ University of Medicine and Pharmacy, 300041 Timișoara, Romania; 8 Department of Oncology, ONCOHELP Hospital, 300239 Timisoara, Romania; 9 Elysee Hospital, 510040 Alba Iulia, Romania; 10 Municipal Hospital, 100337 Ploiești, Romania; 11 Colțea Clinical Hospital, 030167 Bucharest, Romania; 12 Amethyst Radiotherapy Center, 407280 Cluj-Napoca, Romania; 13 ANAPATMOL Research Center, ‘Victor Babes’ University of Medicine and Pharmacy, 300041 Timisoara, Romania

**Keywords:** consensus statement, hormone receptor-positive and human epidermal growth factor receptor 2-negative metastatic breast cancer (HR+/HER2– mBC), triple-negative metastatic breast cancer (mTNBC), Romania, guidelines, Delphi survey

## Abstract

Breast cancer (BC) is the most common malignant disease in women in Romania, with incidence and mortality rates among the highest in Europe. This consensus statement aims to ensure equitable access to care for human epidermal growth factor receptor 2-negative metastatic BC (HR+/HER2– mBC) and triple-negative mBC (mTNBC) in Romania. Between December 2024 and June 2025, a scientific board of 11 oncologists, in collaboration with the Romanian National Society for Medical Oncology (SNOMR), developed national recommendations based on ESMO/NCCN/ABC guidelines, clinical expertise, and local conditions. A modified Delphi survey was conducted among medical oncologists to evaluate acceptance of recommendations with greatest clinical impact. Key recommendations included: mandatory biopsy at metastasis with ER/PgR/HER2 retesting, HER2-low assessment, and molecular profiling (*BRCA*, PIK3CA, AKT1/PTEN, *ESR1*, plus PD-L1 testing in mTNBC); for HR+/HER2– mBC, first-line endocrine therapy plus CDK4/6 inhibitor, followed by targeted agents, chemotherapy, or antibody–drug conjugates based on progression and visceral crisis; for mTNBC, first-line immune checkpoint inhibitor plus chemotherapy in PD-L1-positive, PARP inhibitors in *BRCA*-positive patients, and sacituzumab-govitecan or trastuzumab-deruxtecan later; systematic toxicity monitoring; and integrated supportive and palliative care. Sixty-one oncologists completed the survey, with >90% overall agreement, suggesting broad acceptance of recommendations as Romania’s national standard for mBC care.

## Simple Summary

Breast cancer is the most common malignant disease among Romanian women, and the number of deaths remains among the highest in Europe. This consensus document provides national standards for treating two metastatic types of breast cancer without human epidermal growth factor receptor 2 (HER2) overexpression: hormone receptor-positive/HER2-negative and triple-negative metastatic disease. A scientific board of 11 oncologists, in collaboration with the Romanian National Society for Medical Oncology, adapted international guidelines to fit Romanian realities and then assessed acceptance among medical oncologists through a survey of 61 oncologists. The recommendations include: repeating biopsy and key biomarker tests at metastasis, utilizing modern targeted and immunotherapies when appropriate, carefully monitoring and managing side effects, and providing strong supportive and palliative care. Sixty-one oncologists completed the survey, and an over 90% agreement was achieved, supporting these recommendations as the new national standard.

## 1. Introduction

Breast cancer (BC) remains the most commonly diagnosed cancer worldwide and the leading cause of cancer-related mortality among women [1]. Globally, Europe ranks third in BC incidence, with an age-standardized incidence rate of 75.6 per 100,000 persons per year (over 557,000 new cases) and a mortality rate of 14.6 per 100,000 persons per year (over 144,000 deaths) in 2022 [1].

In Romania, BC is a significant public health issue, with both high incidence and mortality rates and a considerable impact on women’s health [1,2]. While exact annual rates can differ across sources, estimates indicate that BC accounts for approximately 25–30% of all female cancers and 12–13% of all cancers in the country. Over recent years, an increase in BC incidence has been observed among women, with an age-standardized incidence rate of 69.2 per 100,000 persons per year (over 12,000 new cases) being one of the highest in the region in 2022 [2]. Additionally, Romania has one of the highest BC mortality rates in Europe, with over 3800 annual deaths. The estimated annual percentage change in mortality due to BC in Romania is currently +0.2%, thus being the only country in eastern Europe with a growing trend. The lower survival rate than the European average is likely attributed to late diagnosis, when the disease is more challenging to treat, and limited access to advanced treatments in certain regions [1].

A major challenge is the lack of a nationwide population-based screening program. In Romania, the BC screening rate is 10% in women aged 50 to 69, the lowest among European countries (with an average of 65%) [3]. Some regional and private healthcare initiatives are working to tackle this issue by offering screening programs primarily focused on large regional centers. However, these programs are not generally accessible nationwide, particularly in rural and underserved areas [4]. Limited public awareness of BC prevention and early detection also exacerbates the issue. Additionally, the Romanian healthcare system faces inadequate funding, with health spending per capita accounting for 6.5% of the gross domestic product in 2023, as opposed to the European median of 11% [5]. Healthcare labor shortages and disparities between urban and rural services create barriers to timely access and high-quality treatment.

Romania’s BC treatment guidelines emphasize a multidisciplinary approach that follows guidelines adapted from the European Society of Medical Oncology (ESMO) [6] and the National Comprehensive Cancer Network (NCCN) [7], tailored to local resources and regulatory frameworks. For metastatic BC, current standard treatments include chemotherapy (ChT), endocrine therapies (ETs), and targeted therapies (TTs), such as cyclin-dependent kinase 4 and 6 (CDK4/6) inhibitors, phosphatidylinositol-4,5-bisphosphate 3-kinase (PI3K) inhibitors, poly(ADP-ribose) polymerase (PARP) inhibitors, anti-programmed death-ligand 1 (PD-L1) immunotherapies, human epidermal growth factor receptor 2 (HER2) inhibitors, and antibody-drug conjugates (ADCs), which have reshaped the treatment landscape, and in selected cases, surgery and/or radiotherapy (RT) [6,7].

Although Romania provides access to standard ChT, ETs, TTs, and immunotherapy, approval and reimbursement for some newer TTs and advanced diagnostic tools can lag behind western European timelines [8]. While CDK4/6, PARP, and HER2 inhibitors are currently available and fully covered by the National Health Insurance House (NHIH) in specific categories of BC patients, other agents such as the mammalian target of rapamycin (mTOR) inhibitor Everolimus, the novel oral selective estrogen receptor degrader (SERD) Elacestrant, the PIK3CA inhibitor Alpelisib, and the AKT inhibitor Capivasertib, are not yet covered. In addition, access to molecular diagnostics is challenging in some cases (e.g., *estrogen receptor 1* [*ESR1*] mutation testing is not reimbursed), which limits tailored therapeutic decisions. However, clinical trials at major oncology centers across the country help to fill this gap partially.

The current regulatory bodies are actively prioritizing improved access to modern treatments through a national strategic plan for cancer control [9]. These efforts aim to enhance earlier detection, provide access to more effective treatments, and increase the use of molecular tests that may impact treatment decisions (e.g., in *ESR1*-mutated patients) and clinical outcomes.

This consensus statement aims to ensure equitable access to care in Romania by providing recommendations for managing hormone receptor-positive and human epidermal growth factor receptor 2-negative metastatic breast cancer (HR+/HER2– mBC) and triple-negative metastatic breast cancer (mTNBC) as of July 2025. It was developed based on the current guidelines, available evidence, national drug access, and reimbursement status of diagnostic tests and therapies in Romania at that time, along with common daily practice and the authors’ personal views and experiences. To evaluate the level of agreement with the recommendations on innovative management approaches for HR+/HER2– mBC and mTNBC, a Delphi survey was conducted among medical oncologists.

## 2. Materials and Methods

### 2.1. Scientific Board and Development of the Consensus Statement

Between December 2024 and June 2025, a scientific board composed of 11 Romanian oncologists, nationally recognized as experts in the field of BC (list of authors) was gathered to prepare the recommendations in collaboration with the National Society for Medical Oncology from Romania (SNOMR). The recommendations detailed here were built on global guidelines and clinical expertise, incorporating key points for the management of HR+/HER2– mBC and mTNBC.

A document summarizing the most recent existing guidelines was shared with the scientific board. The following published international guidelines for metastatic BC were considered: the ESMO Clinical Practice Guideline for the diagnosis, staging, and treatment of patients with mBC [6] and ESMO mBC Living guideline [10], the NCCN v4.2025 United States guidelines [7], and Advanced Breast Cancer (ABC) 6 and 7 international guidelines [11]. Recommendations from these guidelines were assessed for applicability in the Romanian context, considering the registration status of therapeutic agents, reimbursement policies, and daily clinical practice. Each member of the working group provided input on specific sections, which informed the development of the first draft of the general recommendations for management of HR+/HER2– mBC and mTNBC. These recommendations were subsequently reviewed, discussed, and refined by all 11 experts until consensus was reached.

The following topics were covered:

- Diagnosis, biomarker testing, and disease staging;
- Treatment of local and regional recurrence;
- Systemic treatment approach stratified according to line of treatment;
- Monitoring and follow-up;
- Supportive care.

### 2.2. Assessment of Agreement/Disagreement Level Across Medical Oncologists

Following validation of the recommendations by the scientific board members, a modified Delphi method [12], similar to that used in another consensus paper [13], was utilized to assess the level of agreement and disagreement with the proposed recommendations on current approaches for managing HR+/HER2– mBC and mTNBC. The scientific board selected 11 statements considered to have the greatest practical impact. These statements were shared as questions on a 5-point Likert agreement scale. The wording used for each statement is shown in Table 1.

Subsequently, 80 Romanian local oncologists, members of SNOMR who had at least 5 years of experience in oncology (not necessarily completely dedicated to BC patients) were invited to participate in the Delphi survey in order to capture the rate of agreement and disagreement with the proposed statements. The invitation was sent via email on 1 August 2025, and the voting period closed on 31 August 2025. The survey was administered using Google Forms. Participants were provided with an information page describing the study purpose, voluntary participation, data handling, and anonymity. Individual responses were collected anonymously and accessible only to the study team, not to other participants.

Each participant was asked to indicate their level of agreement with each statement by selecting one of five items from the 5-point Likert scale:

- Completely disagree (further assessed as “disagreement”);
- Partially disagree (further assessed as “disagreement”);
- Partially agree (further assessed as “agreement”);
- Agree (further assessed as “agreement”);
- Completely agree (further assessed as “agreement”).

For analysis, responses were grouped into “disagreement” (completely/partially disagree) and “agreement” (partially agree/agree/completely agree). Agreement/disagreement rates were calculated for each item. Consensus was deemed reached if more than 66.6% of responses favored one of the two possible directions, consistent with Miglietta et al. (2023) [13]. In all other instances, consensus was not deemed achieved. Data were descriptively analyzed.

Ethical review and approval were waived because the study consisted of a voluntary, anonymous survey of members of SNOMR represented by healthcare professionals and collected no personally identifiable or sensitive data; completion of the questionnaire was considered informed consent.

## 3. Results

### 3.1. Consensus Statement

The following recommendations were developed and validated by the 11 members of the scientific board.

#### 3.1.1. Diagnosis, Biomarker Testing, and Disease Staging in Patients with Newly Diagnosed or Recurrent HR+/HER2- mBC and mTNBC

A. *Tumor biology assessment:*
  1. A biopsy is recommended to be performed at the initial diagnosis of mBC to confirm histology and reassess tumor biology (estrogen receptor [ER], progesterone receptor [PgR], and HER2); it is mandatory in de novo cases.
  2. Evaluate ER/PgR and HER2 status at least once in the metastatic setting if clinically feasible.
  3. Pathology reports should provide HER2 results as immunohistochemistry (IHC) score (0, 1+, 2+, and 3+) and, when indicated, in situ hybridization (ISH, amplified versus not amplified) consistent with ASCO/CAP guidelines and CAP reporting standards [14,15]. HER2-positive is defined as IHC 3+ or IHC 2+ with ISH amplification; HER2-negative (no overexpression/amplification) is IHC 0/1+ or IHC 2+ with ISH not amplified [14]. For IHC 0, specify 0 (no staining) or 0+ (incomplete faint/barely perceptible membrane staining in ≤10% of tumor cells) [15].
    ▪ 3a. (optional) Where clinically relevant, HER2 expression may also be described using therapy-related descriptors: HER2-zero (IHC 0; no staining), HER2-ultralow (IHC 0+), HER2-low (IHC 1+ or IHC 2+/ISH–), or HER2-positive (IHC 3+ or IHC 2+/ISH+) [14,15].
  4. Systematic reassessment of HER2 status is recommended during the disease course for cases initially classified as HER2-0.
    - The presence of HER2-low status on at least one sample is sufficient for eligibility for trastuzumab-deruxtecan (T-DXd) therapy.
    - Tumors with low ER expression (1–10%) should be categorized as mTNBC, and therefore, patients should be given access to drugs developed or registered for mTNBC. *Note:* this is a locally adapted position consistent with ABC 6/7 guidelines [11] and the expert community in Romania.
B. *Molecular profiling of therapeutically relevant biomarkers for HR+/HER2– mBC:*
  1. Identify actionable mutations, such as germline breast cancer gene (*BRCA)1/2* mutations in HER2– mBC, *PIK3CA* mutation, PIK3CA/AKT1/PTEN-pathway alterations, and *ESR1* mutations in HR+/HER2– mBC.
  2. TTs should be offered based on biomarker testing (e.g., *PIK3CA* mutations for eligibility for alpelisib; and *ESR1* mutations for eligibility for elacestrant therapy, etc.).
C. *Molecular Profiling of therapeutically relevant biomarkers for mTNBC:*
  1. PD-L1 testing (assay-specific) should be performed before initiating first-line (1L) therapy to determine eligibility for immune checkpoint inhibitors (ICIs).
    - 22C3 assay, PD-L1 combined positive score (CPS) ≥10: eligibility for pembrolizumab + ChT;
    - SP142 assay, PD-L1 immune-cell score IC ≥1% of tumor area: eligibility for atezolizumab and nab-paclitaxel.
  2. Germline *BRCA1/2* mutation testing is required before 1L therapy to predict benefit from PARP inhibitors.
  3. If receptor status in metastatic lesions differs from that of the primary tumor, treatment decisions should be discussed in a multidisciplinary meeting, on a case-by-case basis, considering approved therapies for mTNBC, ER+/HER2−, or HER2+ mBC, to identify the most effective treatment for the patient.
  4. Additional genomic profiling (on tumor tissue or ctDNA) may be performed if the results will impact treatment approach or if the patient can access appropriate clinical trials.
  5. Disease staging: a minimum staging workup should include a comprehensive patient history, physical examination, laboratory tests (hematology and biochemistry), and imaging of the chest, abdomen, and bones to assess metastatic spread.
  6. In mTNBC, brain imaging should be considered even in the absence of neurological signs or symptoms. *Note:* brain MRI is preferred for initial staging, but brain CT may be considered as per local access constraints.

#### 3.1.2. Treatment of Locoregional Recurrence (at the Breast or Chest Wall, with or Without Axillary Lymph Node Involvement) in HR+/HER2– mBC and mTNBC Patients

1. Re-biopsy of recurrent lesions should be performed routinely to determine the molecular profile.
2. Local recurrence after breast conserving surgery (BCS):
  - *If no prior RT*: Repeat BCS; consider axillary staging (if no prior axillary lymph node dissection [ALND]), adjuvant RT (if indicated).
  - *If prior RT administered*: Mastectomy; consider axillary staging (if no prior ALND); repeat RT if feasible (based on prior radiation dose and treatment field constraints) and indicated.
3. Local recurrence after prior mastectomy:
  - *If no prior RT*: Surgical resection should be performed, if feasible, consider surgical axillary staging and post-mastectomy RT to the involved adjacent tissues, including the chest wall and regional lymphatics (e.g., supraclavicular region).
  - *If prior RT administered*: surgical resection if feasible, consider surgical axillary staging if no prior ALND; repeat RT if feasible (based on prior radiation dose and treatment field constraints) and indicated.
4. Unresectable chest wall recurrence: RT is the preferred approach in patients without prior RT.
5. In addition to locoregional treatment, all patients should be evaluated for (neo)adjuvant systemic therapy, including:
  - ET in HR+ mBC (either tamoxifen or aromatase inhibitors [AIs] ± ovarian suppression, depending on menopausal status).
  - ChT in HR+ cases with high-risk or TNBC recurrence.

*Note:* “high-risk” refers to patients with large tumors (≥T1c according to the American Joint Committee on Cancer Tumor–Node–Metastasis staging system in TNBC or ≥5 cm in HR+ disease), node-positive disease, high-grade histology, lymphovascular invasion, young age, or a high-risk genomic profile.

#### 3.1.3. Systemic Treatment Approach for HR+/HER2– mBC

Patients with HR+/HER2– mBC stage IV or recurrent disease without visceral crisis should receive ET alone or ET + TTs.

A. Patients eligible for ET:
  1. 1L treatment in ET-sensitive/-naive patients (with de novo stage IV or mBC, and whose disease progressed >1 year of adjuvant ET)
    ○ **Preferred regimen:** AIs + CDK4/6 inhibitors (abemaciclib, palbociclib, or ribociclib in alphabetic order).
      *Clinical Rationale:* Patients without visceral crisis and hormone-dependent disease derive significant benefits in overall survival (OS), progression-free survival (PFS), objective response rate (ORR), and quality of life (QoL), according to clinical studies and the ESMO-Magnitude of Clinical Benefit Scale.
    ○ **Pre- and perimenopausal women** should receive ovarian function suppression in addition to all endocrine-based therapies.
      *Clinical Rationale:* Ovarian ablation or ovarian function suppression with a luteinizing hormone-releasing hormone (LHRH) agonist confers similar efficacy. Although most clinical trials assessing the effects of AI + CDK 4/6 inhibitors primarily enrolled postmenopausal patients, with only a small subset of premenopausal patients undergoing ovarian suppression, current guidelines support a similar treatment approach in premenopausal patients undergoing ovarian ablation/suppression.
      *Key Phase III Clinical Trials:*
    - PALOMA-2: Palbociclib + letrozole has been shown to significantly improve median PFS versus letrozole alone (24.8 versus 14.5 months, respectively) in postmenopausal patients with HR+/HER2- mBC who had not received prior treatment for metastatic disease, whose disease progressed on prior ET [16].
    - MONARCH 3: Abemaciclib + AI (letrozole or anastrozole) has been shown to significantly improve median PFS versus AI alone (median 29.0 versus 14.8 months, respectively) in postmenopausal women with HR+/HER2- mBC; OS was also improved (median 66.8 versus 53.7 months, respectively), but no statistical significance was reached [17].
    - MONALEESA-7: Ribociclib + goserelin + non-steroidal AI or tamoxifen has been shown to improve PFS versus goserelin + non-steroidal AI or tamoxifen (23.8 versus 13.0 months, respectively) in pre- or perimenopausal women with HR+/HER2- mBC [18]; OS benefit observed (70.2% versus 46.0% at 3.5 years, respectively) [19].
    - MONALEESA-2: Ribociclib + letrozole has been shown to improve PFS versus letrozole alone (25.3 versus 16.0 months, respectively) at 26.4 months follow-up in postmenopausal women with de novo disease or late recurrence HR+/HER2- mBC; ORR was also improved (43% versus 29%, respectively) [20]; median 6-year OS was 69.2 versus 54.3 months, respectively.
      Additionally, a large real-world study found no significant OS differences between 1L ribociclib, abemaciclib, and palbociclib combined with an AI for patients with HR+/HER2 mBC aged over 18 years old. The adjusted hazard ratio (with 95% confidence interval) was 0.98 (0.87–1.10) for ribociclib versus palbociclib (*p* = 0.7531), 0.95 (0.84–1.08) for abemaciclib versus palbociclib (*p* = 0.4292), and 0.97 (0.82–1.14) for abemaciclib versus ribociclib (*p* = 0.6956) [21].
  2. 1L treatment in patients with primary or secondary ET-resistance (whose disease progressed while on adjuvant ET or ≤12 months after the end of ET)
    ○ **Preferred regimen:** Fulvestrant + CDK4/6 inhibitors (abemaciclib, palbociclib, or ribociclib in alphabetic order).
      *Clinical Rationale:* Fulvestrant combined with a CDK4/6 inhibitor is recommended for patients with HR+/HER2– mBC recurring while on adjuvant ET or within 12 months after the end of adjuvant ET, since it is associated with substantial PFS and OS benefits, as well as maintained or improved QoL.
    ○ **Pre- and perimenopausal women** must receive ovarian function suppression in addition to all endocrine-based therapies.
      *Clinical Rationale:* ovarian ablation or ovarian function suppression with an LHRH agonist confers similar efficacy.
    ○ **Alternative:**
      - Palbociclib + inavolisib + fulvestrant in PIK3CA-mutated patients progressing during adjuvant AI;
      - ET alone in highly selected cases (e.g., elderly, multiple comorbidities, low metastatic disease burden): tamoxifen or AI or fulvestrant alone;
      - Consider enrollment in clinical trials, if available.
    *Note:* discuss ovarian ablation in patients remaining pre-menopausal under LHRH agonist.
  3. 2L and subsequent treatment in postmenopausal patients and premenopausal patients with ovarian ablation/suppression with HR+/HER2–, recurrent/stage IV BC:
    ○ **Preferred regimen:** Fulvestrant + CDK4/6 inhibitors (if not used in 1L) in patients progressing during AI-based therapy, with or without prior ChT line.
      *Key Phase III Clinical Trials:*
      - PALOMA-3: Palbociclib + fulvestrant has been shown to significantly improve median PFS versus fulvestrant alone (9.5 versus 4.6 months, respectively) in postmenopausal patients whose disease progressed on prior ET [22].
      - MONARCH 2: Abemaciclib + fulvestrant has been shown to significantly improve median PFS versus fulvestrant alone (16.4 versus 9.3 months, respectively), regardless of menopausal status in patients whose disease progressed on prior ET; 4-year OS also improved (46.7 versus 37.3 months, respectively) [23].
      - MONALEESA-3: Ribociclib + fulvestrant has been shown to improve median PFS versus fulvestrant alone (20.5 versus 12.8 months, respectively) in postmenopausal women who had received up to one line of prior ET for advanced disease [24]; at 42 months follow-up, median PFS was 33.6 versus 19.2 months, respectively, in the 1L subgroup, while in the 2L subgroup was 14.6 and 9.1 months, respectively; overall OS benefit observed (57.8% versus 45.9%, respectively, at 42 months), with similar outcomes in 1L and 2L subgroups [25].
    ○ **TT:** to be offered to patients with actionable mutations and if the corresponding drug is available
      - Oral SERD (elacestrant) for ESR1-mutated patients;
      - inhibitor (alpelisib) for PIK3CA-mutated patients;
      - AKT inhibitor (capivasertib) for patients with PIK3CA-AKT1-PTEN pathway alteration.
      *Key Phase III Clinical Trials:*
      - EMERALD (ESR1-mutant cohort): elacestrant has been shown to significantly improve 12-month PFS versus ET alone (26.8% versus 8.2%, respectively) in patients who had received one-two lines of ET [26].
      - SOLAR-1 (PIK3CA-mutant cohort): alpelisib + fulvestrant has been shown to significantly improve PFS versus fulvestrant alone (11.0 versus 5.7 months, respectively) in patients who had received ET previously [27]; OS also improved (39.3 versus 31.4 months, respectively) [28].
      - CAPItello-291 (AKT pathway-altered population): capivasertib + fulvestrant has been shown to significantly improve median PFS (7.3 versus 3.1 months, respectively) regardless of previous exposure to CDK4/6i and menopausal status; estimated OS at 18 months was 73.2% versus 62.9%, respectively [29].
    ○ **Alternative:**
      - Oral ChT (capecitabine or vinorelbine);
      - Single-agent ChT anthracyclines (epirubicin, doxorubicin and liposomal doxorubicin) or taxanes (paclitaxel, docetaxel);
      - Fulvestrant alone;
      - Trastuzumab deruxtecan (T-DXd) for HER2-low patients;
      - Consider enrollment in clinical trials, if available.
    ○ **Special Considerations**
      - Elderly and frail patients: treatment intensity should be adjusted based on biological age, comorbidities, and functional status rather than chronological age; less aggressive therapeutic regimens may be appropriate in selected cases.
      - Male BC: the selection of other systemic therapies is similar to that of premenopausal women.
B. *Systemic treatment strategy in patients with visceral crisis and/or endocrine refractory*
  1. 1L treatment for *BRCA1/2*-mutated patients:
    ○ **Preferred regimen:** intravenous ChT preferred administration in visceral crisis until progression or unacceptable toxicity.
      *Clinical Rationale*: visceral crisis is a fast-progressing clinical scenario and can benefit from ChT
    ○ PARP inhibitors (olaparib, talazoparib), if available, and previously exposed to ChT in (neo)adjuvant setting.
    ○ **Alternative:**
      - T-DXd for HER2-low/ultralow patients;
      - Ribociclib for premenopausal patients with clinically aggressive disease.
  2. 1L treatment for *BRCA* wildtype patients:
    ○ **Preferred regimen:** ChT in visceral crisis or endocrine resistance (taxanes, capecitabine, vinorelbine, eribulin).
      *ChT options:*
      - Preferred regimen: single-agent ChT (docetaxel, paclitaxel, nab-paclitaxel, doxorubicin, epirubicin, liposomal doxorubicin, capecitabine, vinorelbine, gemcitabine, platinum);
      - Combination ChT is preferred in cases of imminent organ failure.
      *Clinical Rationale:* visceral crisis is a fast-progressing clinical scenario and patients can benefit from chemotherapy.
    ○ **Alternative:** Consider enrollment in clinical trials, if available.
C. *2L and subsequent lines of treatment*
    ○ **Preferred regimen:**
      - T-DXd for HER2-low patients;
      - Sacituzumab govitecan (SG) for patients not suitable for T-DXd (in 3L or later lines).
*Key Phase III Clinical Trials:*
    - DESTINY-Breast04: T-DXd has been shown to significantly improve median PFS versus physician’s choice ChT (9.9 versus 5.1 months, respectively) in patients with HER2-low disease; OS also significantly improved (23.4 versus 16.8 months, respectively) [30].
    - DESTINY-Breast06: T-DXd proved an improvement in median PFS versus physician’s choice ChT (13.2 versus 8.1 months, respectively) in patients with HER2-low disease; in a prespecified exploratory analysis in HER2-ultralow population, the PFS was also improved with T-DXd versus ChT (13.2 versus 8.3 months) [31].
    - TROPiCS-02: SG has been shown to improve median PFS versus ChT (5.5 versus 4.0 months, respectively) and PFS at 12 months (21% versus 7%, respectively) [32].
    ○ **Alternative regimens:** systemic single-agent ChT (eribulin, gemcitabine, carboplatin, pegylated liposomal doxorubicin) or combination of bevacizumab + taxane/capecitabine.
    ○ **Combination regimens useful in certain circumstances:** doxorubicin/cyclophosphamide (AC), epirubicin/cyclophosphamide (EC), cyclophosphamide/methotrexate/fluorouracil (CMF), carboplatin + gemcitabine, paclitaxel + gemcitabine, docetaxel + capecitabine, etc.

Once the visceral crisis is managed and the patient’s condition is stabilized, the treatment plan can be reassessed, and maintenance therapy with ET or TT should be considered.

**Summary of Consensus Model for HR+/HER2- mBC**

**Diagnosis and biomarker testing**: actionable biomarkers (BRCA, ESR1, PIK3CA, PTEN, AKT1) testing preferred from the diagnosis or at least at the progression of disease.

**1L treatment**: ET and CDK4/6 inhibitors as standard; consider T-DXd for fast progressors (if HER2-low) and either ChT or PARP inhibitors after ChT in (neo)adjuvant setting in patients with visceral crisis or endocrine refractory tumors.

**2L treatment**: Fulvestrant and CDK4/6i if not used before, ET and TT in specific targets, ChT, ADCs.

#### 3.1.4. Systemic Treatment Approach for mTNBC

A. *1L treatment*
  1. PD-L1+ patients, regardless of *BRCA* mutation status:
    ○ **Preferred regimen:** Immune checkpoint inhibitor (ICI) + ChT
      - Pembrolizumab + paclitaxel, nab-paclitaxel, or carboplatin–gemcitabine if PD-L1 CPS ≥10 as assessed by 22C3 CPS assay;
      - Atezolizumab + nab-paclitaxel if PD-L1 ≥1% as assessed by SP142 assay.
*Key phase III clinical trials:*
    - Keynote-355: 1L pembrolizumab + investigator’s choice ChT (paclitaxel, nab-paclitaxel, or carboplatin–gemcitabine) has been shown to improve PFS versus ChT in patients with mTNBC PD-L1 CPS ≥10 (9.7 versus 5.6 months, respectively); median OS was also significantly improved (23.0 versus 16.1 months, respectively) [33].
    - IMpassion130: Atezolizumab + nab-paclitaxel has been shown to significantly improve median PFS versus nab-paclitaxel alone (7.5 versus 5.0 months, respectively) in PD-L1-positive mTNBC patients; median overall OS was 25.0 versus 15.5 months, respectively [34].
  2. PD-L1– and *BRCA*+ patients:
    ○ **Preferred regimen:** PARP inhibitor (olaparib) but not recommended for “de novo” patients. Prior treatment with anthracyclines and/or taxanes in a (neo)adjuvant setting is mandatory.
*Key phase III clinical trial:*
    - OlympiAD: Olaparib therapy has been shown to significantly improve PFS versus investigator’s choice single-agent ChT (7.0 versus 4.2 months, respectively) in *BRCA*-mutated patients with HER2– mBC who received ≤2 lines of ChT [35]; median overall OS was 19.3 months for olaparib and 17.1 months for ChT, and 3-year survival rate was 27.9% versus 21.2%, respectively; in 1L treatment, median OS was longer with olaparib versus ChT (22.6 versus 14.7 months, respectively), and 3-year survival rates improved (40.8% versus 12.8%, respectively) [36].
  3. If PD-L1– and *BRCA* wild-type patients:
    ○ **Preferred regimen:** ChT (single-agent or combination)
      - ChT options—taxanes (paclitaxel, docetaxel), anthracyclines (epirubicin, doxorubicin, pegylated liposomal doxorubicin), or platinum-based chemotherapy (carboplatin, cisplatin);
      - In case of imminent organ failure, combination therapy is preferred based on a taxane and/or anthracycline combination ± bevacizumab (first line only) if available.
*Key phase III clinical trial:*
    - Alliance: 1L nab-paclitaxel + bevacizumab resulted in a median PFS of 7.4 months, and paclitaxel + bevacizumab in a median PFS of 11 months in patients with locally recurrent or mBC [37].
B. *2L Treatment*
  1. For *BRCA1/2+* patients:
    ○ **Preferred regimen:** PARP inhibitors (olaparib or talazoparib)
    *Clinical Rationale*: *BRCA*+ tumors are sensitive to PARP inhibitors due to defective DNA repair mechanisms.
    ○ **Alternative:** platinum-based ChT (if not used as 1L therapy).
  2. For *BRCA* wild-type patients:
    ○ **Preferred regimen:** Trop-2 ADC (SG).
      *Key phase III clinical trial:*
    - ASCENT: SG has been shown to significantly improve median PFS versus ChT (5.8 versus 1.6 months, respectively) in patients with mTNBC without brain metastases who previously received 2 or 3 lines of treatment; median OS was also significantly improved (12.2 versus 6.7 months, respectively) [38]. Both PFS and OS were significantly improved regardless of Trop-2 expression [39].
    ○ **Alternative:** other ADCs or chemotherapy regimens based on prior treatment exposure and patient health.
C. *3L treatment and Beyond*
    ○ **Preferred regimen: ADCs:**
      - SG is the primary ADC used for heavily pretreated mTNBC patients.
      - T-DXd is an alternative for patients with HER2-low.
*Key Phase I/II single-group clinical trial:*
    - IMMU-132-01: Response rate to SG was 34.3% and median duration of response was 9.1 months in patients with refractory mTNBC as assessed by an independent central review committee; median PFS was 5.5 months, and OS was 13.0 months [40].
*Key phase III clinical trial:*
    - ASCENT: SG has been shown to significantly improve PFS versus ChT (5.6 versus 2.5 months, respectively) in patients with mTNBC without brain metastases who previously received >3 lines of treatment; OS was also significantly improved (12.1 versus 7.1 months, respectively) [38]. Both PFS and OS were significantly improved, regardless of Trop-2 expression [39].
    - Destiny Breast04: in a subgroup of 58 HR–mTNBC patients, improved PFS and OS were observed with T-DXd versus physician’s choice ChT (8.5 versus 2.9 months for PFS, respectively, and 18.2 versus 8.3 months for OS, respectively) [30].
    ○ **Chemotherapy:**
      - Monotherapy with eribulin, vinorelbine, gemcitabine, or other agents not previously used in prior lines of therapy;
      - Sequential single-agent chemotherapy is preferred to combination chemotherapy to minimize toxicity.
    ○ **Alternative:** consider enrollment in clinical trials, if available.
  *Note:* patients with metastatic disease and limited treatment options should be referred for clinical trials, particularly those exploring novel ADCs, bispecific antibodies, and next-generation immunotherapy.

**Summary of Consensus Model for mTNBC**

**Diagnosis and Biomarker Testing:** Molecular profiling, PD-L1 and *BRCA* mutation testing, and liquid biopsy.

**1L Treatment:** ICIs + ChT for PD-L1+ patients; ChT for PD-L1– *BRCA*-wild-type patients, PARP inhibitors for PD-L1– *BRCA*-mutated patients.

**2L Treatment:** SG for most patients; PARP inhibitors for *BRCA*-mutated patients (if not used previously).

**3L and Beyond:** SG, investigational ADCs, and chemotherapy.

#### 3.1.5. Monitoring and Toxicity Management During HR+/HER2– mBC and mTNBC Treatment

Monitoring disease progression, assessing treatment response, and managing toxicities are critical.

A. *Imaging:*
  Use computed tomography (CT), magnetic resonance imaging (MRI), or positron emission tomography-computed tomography (PET-CT) every 12 weeks or more frequently if the clinical status indicates progression.
  ○ CT of the thorax, abdomen, and pelvis, MRI of the brain, and suspected bone lesions that require further evaluation are recommended. PET-CT may serve as an additional assessment tool when necessary.
  ○ For patients with bone-only disease, a bone scan can be used for evaluation every 3–6 months; however, it should be combined with visceral imaging evaluation.
B. *Toxicity Management:*
  Close monitoring is essential for managing the toxicities associated with ChT, AIs, ICIs, and ADCs.
  ○ **T-DXd:** Regular monitoring for interstitial lung disease (ILD)/pneumonitis (serious side effect) is recommended. For patients with a history of ILD/pneumonitis, there are no data on managing the safety or toxicity of this drug in a trial.
  ○ **SG:** Monitor for neutropenia, diarrhea, nausea, and use appropriate prophylaxis.
  ○ **ICIs:** Monitor for immune-related adverse events (irAEs) such as pneumonitis, colitis, hepatitis, and endocrine disorders.
  ○ For toxicities related to ET and ChT, please refer to their specific indications for use.

#### 3.1.6. Supportive Care and Patient-Centered Approach

Given the aggressive nature HR+/HER2- mBC and mTNBC, supportive care is essential to improve QoL and should be integrated into the treatment plan from the onset. Early incorporation of palliative care is crucial for optimal symptom control and tolerable therapy administration.

*Key Symptom Management Areas:*

○ **Pain:** ensure access to effective analgesia, including opioids when indicated.
○ **Fatigue:** use individualized non-pharmacological (e.g., exercise) and pharmacological interventions.
○ **Dyspnea:** treat underlying causes; consider oxygen for hypoxemia and medication such as opioids, corticosteroids, opioids, or benzodiazepines.
○ **Nausea/Vomiting:** use antiemetics, corticosteroids, or octreotide and address reversible causes.
○ **Mucositis/Stomatitis:** apply local treatments (e.g., corticosteroid mouthwash, gentle oral care).
○ **Peripheral neuropathy:** consider agents such as duloxetine, gabapentin, pregabalin, or antidepressants.
○ **Hand-Foot syndrome:** employ preventive skin care, protective footwear, and avoidance of friction or heat.
○ **Menopausal symptoms:** prefer non-pharmacological approaches and local therapies for dyspareunia; systemic hormone replacement is generally not recommended.
○ **Sexual Health:** provide routine assessment and counseling.
○ **Cognitive Impairment:** encourage physical activity and manage contributing factors (e.g., pain, fatigue, sleep disorders, mood disturbances, vitamin deficiencies).

**Psychosocial Support:**

○ Patients should have access to psychological and social support services from diagnosis.
○ Discussions on end-of-life preferences should be encouraged with patient consent.

**Patient Education:**

○ Patients should be informed about treatment goals, potential adverse effects, and available support services to enhance adherence and QoL.

**Summary of consensus recommendation:**

Supportive and patient-centered care should prioritize early palliative care integration, symptom management, psychosocial support, and patient education.

### 3.2. Delphi Survey Outcomes

**Figure 1:**
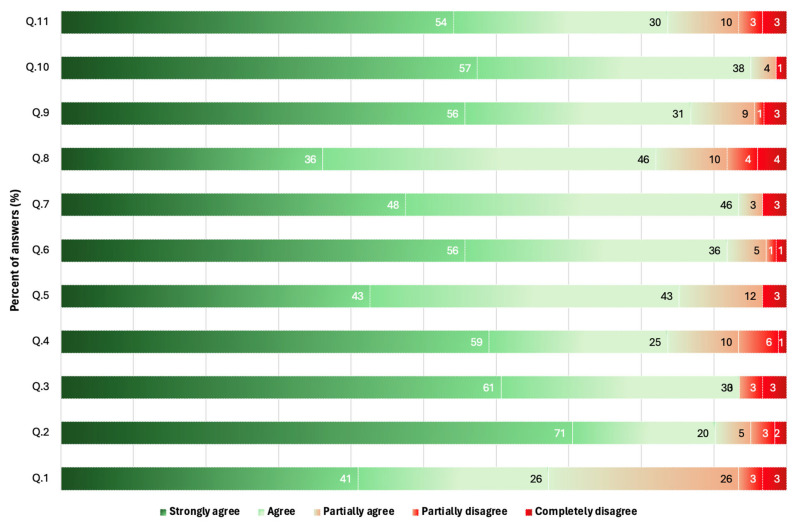
Level of agreement among medical oncologists.

Of 80 specialists or senior physicians in medical oncology invited to participate in the Delphi process, 61completed the survey (76.3% response rate). Because consensus was reached for all recommendations incorporating recent diagnostic and therapeutic updates, with agreement levels exceeding 90%, the process was completed after a single review round (Table 1). Details on the level of agreement among medical oncologists are shown in Figure 1.

## 4. Discussion

The Romanian consensus guideline provides a comprehensive, evidence-based framework for the diagnosis, treatment, and monitoring of HR+/HER2– mBC and mTNBC nationwide. Additionally, the survey results reveal a strong consensus among oncologists, with statements indicating levels of agreement or disagreement with the current strategies for HR+/HER2– mBC and mTNBC management.

### 4.1. Diagnosis and Biomarkers

The approval of new therapeutic agents for tumors with HER2-low expression has created the need to adopt new definitions in clinical practice for interpreting HER2 test results and distinguishing different clinical entities within tumors that do not show HER2 overexpression and/or receptor tyrosine-protein kinase erbB-2 gene amplification [41]. Traditionally, tumors without HER2 overexpression or gene amplification have been classified as HER2-negative according to ASCO/CAP criteria (IHC 0, 1+, or 2+ with ISH not amplified [14]. With the emergence of ADCs, this classification has been further refined using the therapy-relevant subset HER2-low (IHC 1+ or IHC 2+/ISH not amplified) [41]. In addition, expert consensus has proposed sub-stratification within IHC score 0 to distinguish HER2-zero (no staining) from HER2-ultralow, corresponding to IHC 0+ (incomplete, faint/barely perceptible membrane staining in ≤10% of tumor cells) [41]. CAP reporting standards provide a practical framework for documenting this 0 versus 0+ distinction [15]. Accordingly, in this consensus paper, we use ASCO/CAP IHC and ISH results as the primary reporting framework, while “HER2-low” and “HER2-ultralow” are presented as optional therapy-related descriptors when clinically relevant. These classifications have become increasingly relevant with the introduction of ADC agents (i.e., T-DXd and SG) in patient management [42]. The assessment of HER2-low and HER2-ultralow disease is feasible in routine practice using standard immunohistochemistry, but is sensitive to pre-analytical factors, scoring criteria, and inter-observer variability. Therefore, pathology standardization and harmonized IHC protocols are essential to reduce inter-laboratory variability. In Romania, pathology laboratories have implemented HER2-low and HER2-ultralow assessment in routine diagnostics, supporting the feasibility of reliable HER2 classification when standardized procedures are applied. In this context, 93.4% of medical oncologists agreed that systematic reassessment of HER2 status should be performed in the daily clinical practice in patients with newly diagnosed or recurrent HR+/HER2– mBC and mTNBC, particularly when they were initially classified as HER2-zero. Moreover, 95% of participants agreed that when metastatic lesions show discordant HR status compared to the primary tumor, treatment should be individualized through multidisciplinary discussion and guided by approved options for mTNBC, ER+/HER2–, or HER2+ mBC.

Regarding actionable mutations in mBC, 93.5% of participants agree that identifying these mutations remains a key step of personalized therapy. For instance, germline *BRCA1/2* mutations in HER2– disease justify the use of PARP inhibitors, while *PIK3CA* mutations and PIK3CA/AKT1/PTEN pathway alterations open the way for PI3K or AKT inhibitors. Presence of *ESR1* mutations in HR+/HER2– mBC have also become clinically relevant, guiding the use of next-generation ETs (i.e., oral SERDs) [43]. These molecular targets highlight the importance of routine genomic testing to improve treatment customization. However, at the time this consensus was developed, the Romanian NHIH only reimbursed biomarker testing and targeted therapies for patients with *BRCA1/2* mutations. Expanding reimbursement to include *ESR1*, PIK3CA, *PTEN*, *AKT1*, and *PALB2* mutation testing is essential for optimizing personalized treatment strategies. Ensuring broader access to these biomarkers will enhance precision medicine approaches and support better treatment decisions for HR+/HER2– mBC patients.

Regarding the initial diagnosis and staging of mTNBC, the scientific board highlights that PD-L1 expression testing is required before initiating 1L therapy to assess eligibility for ICIs. Additionally, a 93.4% consensus was reached on performing brain imaging to evaluate metastases in mTNBC patients, even in the absence of neurologic signs or symptoms. Similar to the Italian consensus, the Romanian working group advocates for a subtype-oriented approach when deciding to proactively evaluate for brain metastases [13].

### 4.2. Treatment Strategies

In Romania, the management of mBC generally follows international guidelines, such as ESMO and NCCN [6,7,10]. Despite this, access to several innovative therapies is limited or delayed, which influences treatment decisions and may impact patient outcomes.

**HR+/HER2– mBC**

Regarding 1L therapy in HR+/HER2– mBC patients, a 97.2% consensus was reached in favor of NSAIs + CDK4/6 inhibitors (abemaciclib, palbociclib, or ribociclib, in alphabetical order) as the preferred regimen for premenopausal women with ovarian suppression or ablation, as well as for postmenopausal women. Additionally, 96.7% of participants agreed that male patients should receive the same regimen as premenopausal women. Standard ET is widely accessible, and all three CDK4/6 inhibitors are approved by the National Agency for Medicines and Medical Devices of Romania (ANMROs) for this indication and are reimbursed by NHIH. For *PIK3CA*-mutated patients who progress during adjuvant AI, palbociclib + inavolisib + fulvestrant are available through an early access program, providing a strong foundation for 1L therapy.

**Figure 2:**
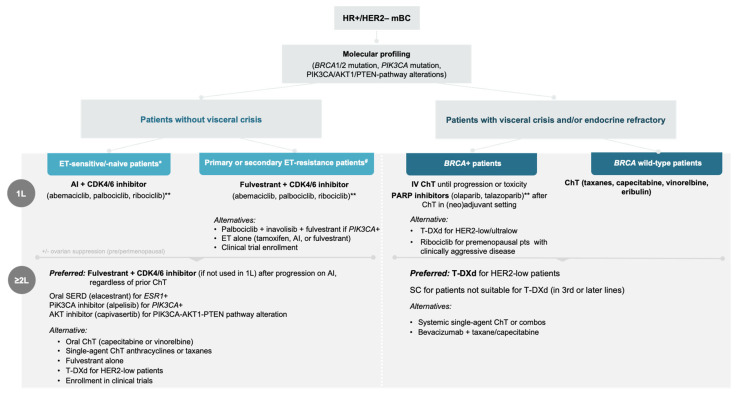
Consensus on systemic treatment approach for HR+/HER2– mBC. * Patients presenting with de novo stage IV or mBC and whose disease progressed > 1 year of adjuvant ET; ** shown in alphabetical order; ^#^ Patients whose disease progresses while on adjuvant ET or ≤12 months after the end of ET. AI, aromatase inhibitor; *BRCA*, breast cancer gene; CDK4/6, cyclin-dependent kinases 4 and 6; ChT, chemotherapy; IV, intravenous; HR+/HER2– mBC, hormone receptor-positive and human epidermal growth factor receptor 2-negative metastatic breast cancer; ET, endocrine therapy; mBC, metastatic breast cancer; PARP, poly-ADP ribose polymerase; SERD, selective estrogen receptor degrader; SG, sacituzumab govitecan; T-DXd, trastuzumab deruxtecan; 1L, first line; ≥2L, second and subsequent lines of treatment.

The 2L metastatic HR+ setting remains a significant challenge in the Romanian clinical practice. For patients with HR+/HER2– mBC who are progressing on AI-based therapy, with or without prior ChT, the scientific board unanimously agreed that the preferred option is fulvestrant + CDK4/6 inhibitor, provided this class has not been used in the 1L setting. In cases with actionable mutations, TTs should be considered: elacestrant for *ESR1*-mutated patients, alpelisib for *PIK3CA*-mutated patients, and capivasertib for patients with PIK3CA–AKT1–PTEN pathway alterations. Currently (as of Jan 2026), testing for *ESR1* mutations is not reimbursed in Romania, nor are the targeted agents elacestrant, alpelisib, and capivasertib. Although these therapeutic options may be available in some compassionate use or early access programs at the time of this consensus, limited access may lead clinicians to rely on less effective therapies or delay the use of these agents, potentially impacting disease control and patient outcomes. Additional treatment options include oral ChT (capecitabine, vinorelbine), single-agent ChT with anthracyclines (epirubicin, doxorubicin, liposomal doxorubicin) or taxanes (paclitaxel, docetaxel), or fulvestrant monotherapy. Everolimus + AI (exemestane) could also be used after recurrence or progression following an NSAI, but it is not reimbursed in Romania, which limits its access and use. For patients with HER2-low and HER2-ultralow disease, T-DXd is considered the preferred option in the 2L setting based on the robust efficacy demonstrated in DESTINY-Breast06 trial [31]. However, T-DXd is not yet reimbursed in Romania. SG may be used in later lines, particularly for patients who are not suitable candidates for T-DXd, with most evidence supporting its role in the 3L or subsequent settings [32]. The therapeutic approach is summarized in Figure 2.

In HR+/HER2– mBC patients with visceral crisis or endocrine-refractory, the Romanian scientific board agreed that intravenous ChT is the preferred 1L. In cases with *BRCA1/2* mutations, ChT remains the initial choice, and PARP inhibitors (olaparib) may be considered in later lines (Figure 2). For *BRCA* wild-type patients, single-agent ChT with taxanes, capecitabine, vinorelbine, or eribulin is preferred, and enrollment in clinical trials should be encouraged where feasible. In 2L and subsequent lines, ADCs play a central role, and 91.8% of medical oncologists agreed that once the visceral crisis is managed, the maintenance therapy with ET or TT should be considered. The 8.2% level of disagreement among oncologists may reflect the challenges faced when considering the optimal sequencing of ADCs in daily clinical practice. Selection of treatment should be based not only on the existence of targetable mutations but also on clinical evidence of safety and effectiveness [44].

**mTNBC**

For PD-L1–positive mTNBC, the preferred 1L strategy agreed by the scientific board is the combination of an ICI + ChT. Specifically, pembrolizumab combined with paclitaxel, nab-paclitaxel, or carboplatin–gemcitabine is recommended for patients with PD-L1 ≥10 (as assessed by 22C3). Alternatively, atezolizumab in combination with albumin-bound paclitaxel is an option for patients with PD-L1 ≥1% (as assessed by SP142). Although all ChT agents are accessible in Romania, and immunotherapy is reimbursed for 1L treatment, only paclitaxel and nab-paclitaxel ChT are covered when combined with pembrolizumab, which limits access to other options.

For PD-L1-negative, *BRCA* wild-type mTNBC, a 95.6% consensus was reached in favor of ChT as the preferred regimen, either as a single agent or in combination. Recommended options include taxanes (paclitaxel, docetaxel), anthracyclines (epirubicin, doxorubicin, pegylated liposomal doxorubicin), or platinum-based agents (carboplatin, cisplatin). In cases of imminent organ failure, combination ChT is preferred, typically involving a taxane and/or anthracycline backbone, with the optional addition of bevacizumab in the 1L setting when available. In Romania, bevacizumab reimbursement is limited to use with paclitaxel or capecitabine in the 1L setting, only when alternative therapies are not appropriate.

In the 2L setting, systemic treatment selection is primarily guided by *BRCA* mutation status. For *BRCA1/2+* patients, there was strong consensus (98.6%) supporting PARP inhibitors (olaparib or talazoparib) as the preferred option, with platinum-based ChT considered an alternative in those not previously exposed. However, in Romania, olaparib is currently the only PARP inhibitor available. For *BRCA* wild-type patients, consensus was also high (93.4%), with SG as the preferred 2L therapy. Similar to the level of disagreement observed in HR+/HER2– mBC, the 6.6% disagreement recorded for the selection of 2L therapy in this category of patients reflects the challenges faced when considering ADCs sequencing. Current clinical trial evidence is limited, coming from the ASCENT trial, in which TNBC patients received SG after ChT in 2L or later [38,39].

**Figure 3:**
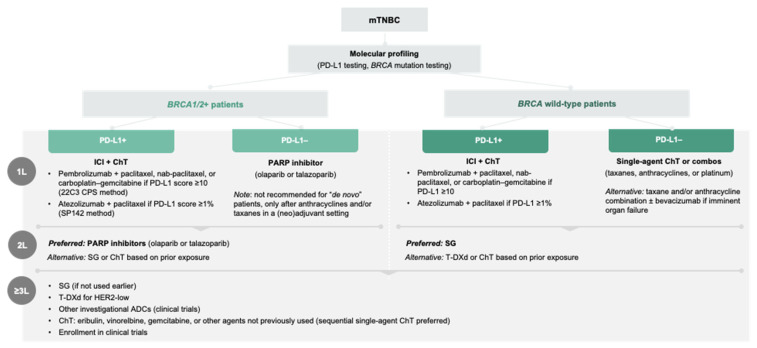
Consensus on systemic treatment approach for mTNBC. ADC, antibody-drug onjugates; *BRCA*, breast cancer gene; ChT, chemotherapy; ICI, immune checkpoint inhibitor; HER2, human epidermal growth factor receptor 2; mTNBC, metastatic triple negative breast cancer; PARP, poly-ADP ribose polymerase; PD-L1, anti-programmed death-ligand 1; SG, sacituzumab govitecan; T-DXd, trastuzumab deruxtecan; 1L, first line; 2L, second; ≥3L, third and subsequent lines of treatment.

The therapeutic approach in mTNBC is summarized in Figure 3.

### 4.3. ADCs Sequencing in Patients with HER2-Low Disease

The recent introduction of the HER2-low concept has added complexity to treatment decision-making, as both SG and T-DXd are now options for patients with metastatic or advanced TNBC and HR+ HER2-low disease. Currently, the optimal choice and sequencing of these ADCs remain unclear, because clinical trial evidence is limited to several studies: DESTINY-Breast04 trial, in which patients with HER2-low disease received T-DXd after 1L to 2L ChT [30], DESTINY-Breast06 trial, in which patients with HR+/HER2-low and HER2-ultralow mBC received T-DXd after ≥1L ET and no ChT [31], TROPiCS-02 trial, in which HR+/HER2– mBC patients received SG after 2L to 4L ChT [32], and ASCENT trial, in which TNBC patients received SG after ChT in 2L or later [38,39].

In the NCCN v4.2025 guidelines, SG is listed as a Category 1, preferred regimen in the 2L setting for mTNBC, whereas T-DXd is positioned as the preferred ADC for HER2-low disease (HR+ or TNBC) [7]. In contrast, the ABC guidelines recommend SG as the preferred regimen for patients with mTNBC who have previously received ≥2L of therapy, including at least one line in the metastatic setting [11]. This is mainly because clinical trial data for T-DXd in TNBC are limited to the DESTINY-Breast04 trial, which enrolled only 58 patients with TNBC and 52 patients with HER2-low, and the positive signal was derived from an exploratory subgroup analysis rather than a TNBC/HER2-low cohort [45]. Moreover, no head-to-head clinical trials comparing SG and T-DXd exist on the optimal sequencing of ADCs. Emerging reports suggest potential cross-resistance and diminished efficacy when used sequentially, as both ADCs share a topoisomerase I inhibitor payload [46]. A study involving mBC patients with HR+/HER2–, TNBC, HER2 low who received more than one ADC (with a median of 4 prior treatment lines) before starting a second ADC found that the median PFS on the 1st ADC was significantly longer at 7.55 months, compared to 2.53 months on the 2nd ADC (*p* = 0.006), highlighting that a subset of patients develops cross-resistance to ADC after initial treatment [47].

Several real-world studies have attempted to address this knowledge gap in the sequencing of ADCs when administered as 2L or in subsequent lines. Among these, a retrospective study (N = 179) conducted in France in HR+ or HR–/HER2-low patients who received sequential ADC therapy reported modest outcomes with sequential ADC therapy, with a median PFS on the second ADC (PFS2) of 2.7 months [48].

PFS is generally lower for the ADC used subsequently, regardless of HR status or the order of administration [48,49], highlighting the importance of selecting the initial ADC based on robust clinical trial evidence to maximize patient benefit. At present, definitive prospective data to guide optimal sequencing of ADCs are lacking; therefore, recommendations will require periodic updates as prospective and real-world evidence emerges. Future research should focus on defining optimal sequencing strategies, identifying biomarkers predictive of response and resistance, and developing combination approaches to mitigate potential cross-resistance between ADCs. Such efforts will be crucial in informing clinical decisions and maximizing the therapeutic benefits of ADCs for patients with HER2-low mBC.

### 4.4. Supportive Care and Patient-Centered Approach

In Romania, the management of patients with HR+/HER2– mBC and mTNBC is further complicated by “medical deserts”, where limited personnel, under-resourced facilities, long waiting times, high treatment costs, gaps in palliative and supportive care, and socio-cultural barriers restrict access to timely cancer care [50]. Although by 2015, Romania had developed 115 specialized palliative care services, including inpatient, outpatient, and home-based options [51], access remained limited and uneven across regions, with a shortage of professionals trained in symptom control and end-of-life care. Continuous efforts are being made to strengthen palliative medicine training [52]. Psychological support also remains suboptimal, with too few psycho-oncologists and limited coordination with oncology teams, leading to delays in assessment and intervention. Expanding access to psycho-oncology services and strengthening social support networks for patients and their families are essential for alleviating the emotional and financial burden of BC patients. Addressing these gaps is crucial for complementing systemic anticancer therapy, especially given the aggressive nature of mTNBC and the chronic course of HR+/HER2– disease, where integrated palliative and psychosocial support greatly influences QoL and adherence to treatment.

Potential improvements include updating clinical protocols, expanding palliative care, strengthening prevention programs, fostering multidisciplinary collaboration, developing patient support and education programs. However, significant obstacles remain, especially the shortage of oncology specialists, regional disparities in cancer care, and limited access to modern therapies in rural areas [50].

## 5. Conclusions

This national consensus combines international guideline standards with national access considerations and expert opinion, providing structured recommendations for high-quality care in patients with HR+/HER2– mBC and mTNBC in Romania. These recommendations were tailored to the realities of drug availability and reimbursement in the country. Delphi survey outcomes confirmed a high level of consensus among medical oncologists, supporting the integration of these recommendations into routine clinical practice.

For HR+/HER2– mBC, biomarker-driven therapeutic approaches are emphasized from diagnosis, with ET plus CDK4/6 inhibitors as the preferred first-line therapy, followed by fulvestrant- or target-based strategies in the second line, and chemotherapy, ADCs, or participation in clinical trials in third or later lines of treatment (Figure 2). In mTNBC, PD-L1 and *BRCA* testing are recommended, with ICIs plus chemotherapy for PD-L1+ patients, SG as the preferred second line option, and PARP inhibitors for *BRCA*+ patients. Beyond the second line, ADCs, chemotherapy, and investigational agents are preferred (Figure 3). Across both subtypes, supportive care, psychosocial interventions, and patient-centered education are needed to improve patient outcomes.

## Figures and Tables

**Table 1:** Delphi consensus outcomes.

Theme	Statement		Consensus *N* = 61
Tumor biology	**Q.1**	In patients with newly diagnosed or recurrent HR+/HER2– mBC and mTNBC, systematic reassessment of HER2 status during disease course is recommended for cases initially classified as HER2 0. The presence of HER2-low status on at least one sample is sufficient for eligibility for T-DXd therapy. Tumors with low ER expression (1–10%) should be categorized as mTNBC, and therefore, patients should be given access to drugs developed or registered for mTNBC. *	93.4%
	**Q.2**	If HR status in metastatic lesions differs from the primary tumor, treatment decisions should be discussed in the multidisciplinary meeting, on a case-by-case basis, considering approved therapies for mTNBC, ER+/HER2−, or HER2+ mBC, to identify the most effective treatment for the patient.	95.1%
Molecular profiling	**Q.3**	Identify actionable mutations, such as germline *BRCA1/2* mutations in HER2– mBC, *PIK3CA* mutation, PIK3CA/AKT1/PTEN-pathway alterations, and *ESR1* mutations in HR+/HER2– mBC.	93.5%
Disease staging	**Q.4**	A minimum staging workup should include comprehensive patient history, physical examination, laboratory tests (hematology and biochemistry), and imaging of the chest, abdomen, and bones to assess metastatic spread. In mTNBC brain imaging should be considered even in absence of neurologic signs or symptoms.**	93.4%
Therapeutic approach	**Q.5**	Patients with HR+/HER2– mBC and mTNBC and locoregional recurrence ***, in addition to locoregional treatment, should be evaluated for (neo)adjuvant systemic therapy, including: ET in HR+ disease; ChT in high-risk or TNBC recurrence.	96.7%
	**Q.6**	1L systemic treatment approach in premenopausal patients undergoing ovarian ablation/suppression or postmenopausal women with HR+/HER2– mBC: Preferred regimen: AIs + CDK4/6 inhibitors. Alternatives: - Fulvestrant + CDK4/6 inhibitors for ER+ patients progressing on ET; - Palbociclib + inavolisib + fulvestrant for patients with *PIK3CA* mutation and progression during adjuvant AI; - T-DXd in HER2-low patients progressing within 6 months after adjuvant ChT; - ET alone in highly selected cases (e.g., elderly, multiple comorbidities, low metastatic disease burden): tamoxifen or AI or fulvestrant alone; - Consider enrollment in clinical trials if available.	97.2%
	**Q.7**	In male patients with HR+/HER2– mBC, 1L systemic treatment selection of other systemic therapies is similar to that of premenopausal women.	96.7%
	**Q.8**	2L and subsequent systemic treatment approach in HR+/HER2– mBC patients with visceral crisis or endocrine refractory: Once the visceral crisis is managed and the patient’s condition is stabilized, the treatment plan can be reassessed, and maintenance therapy with ET or TT should be considered.	91.8%
	**Q.9**	1L systemic treatment approach for mTNBC, PD-L1– and *BRCA wild-type* patients: Preferred regimen ChT (single-agent or combination). - Options: taxanes (paclitaxel, docetaxel), anthracyclines (epirubicin, doxorubicin, pegylated liposomal doxorubicin), or platinum-based ChT (carboplatin, cisplatin). In case of imminent organ failure, combination therapy is preferred based on a taxane and/or anthracycline combination ± bevacizumab (1L only) if available.	95.6%
	**Q.10**	2L systemic treatment approach in mTNBC, *BRCA1/2*-mutated patients: Preferred regimen: PARP inhibitors (olaparib or talazoparib); Alternative: Platinum-based ChT (if not used as 1L).	98.6%
	**Q.11**	2L systemic treatment approach in mTNBC, *BRCA-wild type* patients Preferred regimen: Trop-2 ADC (SG).	93.4%

Note: Percentages indicate the total proportion of answers considered as “agreement” (sum of partially agree, agree, and completely agree). * This is a locally adapted position consistent with ABC 6/7 guidelines [11] and the expert community in Romania. ** Brain MRI is preferred for initial staging, but brain CT may be considered as per local access constraints. *** At the breast or chest wall, with or without axillary lymph node involvement. 1L, first line; 2L, second line; ADC, antibody-drug conjugate; AIs, aromatase inhibitors; *BRCA*, breast cancer gene; CDK4/6, cyclin-dependent kinases 4 and 6; ChT, chemotherapy; ER, estrogen receptor; *ESR1*, estrogen receptor 1 gene; ET, endocrine therapy; HR+/HER2– mBC, hormone receptor-positive and human epidermal growth factor receptor 2-negative metastatic breast cancer; mTNBC, metastatic triple negative breast cancer; N, number of participants; PARP, poly-ADP ribose polymerase; PD-L1, Programmed Cell Death Ligand; SG, sacituzumab govitecan; T-DXd, trastuzumab deruxtecan.

## Data Availability

The data presented in this study are available on request from the corresponding author.

## Abbreviations

The following abbreviations are used in this manuscript:

1L	First Line
2L	Second Line
≥3L	Third and Subsequent Lines of Treatment
ABC	Advanced Breast Cancer
ADC	Antibody-Drug Conjugate
AI	Aromatase Inhibitor
ALND	Axillary Lymph Node Dissection
ANMRO	National Agency for Medicines and Medical Devices of Romania
ASCO/CAP	American Society of Clinical Oncology/College of American Pathologists
BC	Breast Cancer
BCS	Breast Conserving Surgery
*BRCA*	*Breast Cancer Gene*
CDK4/6	Cyclin-Dependent Kinases 4 and 6
ChT	Chemotherapy
CT	Computed Tomography
ctDNA	Circulating Tumor DNA
ER	Estrogen Receptor
ESMO	European Society of Medical Oncology
*ESR1*	*Estrogen Receptor 1* Gene
ET	Endocrine Therapy
HER2	Human Epidermal Growth Factor Receptor 2
HR-	Hormone Receptor Negative
HR+	Hormone Receptor Positive
HR+/HER2– mBC	Hormone Receptor-Positive and HER2-negative Metastatic Breast Cancer
ICI	Immune Checkpoint Inhibitor
IHC	Immunohistochemistry
LHRH	Luteinizing Hormone-Releasing Hormone
MRI	MRI: Magnetic Resonance Imaging
mBC	Metastatic Breast Cancer
mTNBC	Metastatic Triple-Negative Breast Cancer
NCCN	National Comprehensive Cancer Network
NSAI	Nonsteroidal Aromatase Inhibitor
OS	Overall Survival
PARP	Poly-ADP Ribose Polymerase
PET-CT	Positron Emission Tomography—Computed Tomography
PgR	Progesterone Receptor
PI3K	Phosphatidylinositol-4,5-bisphosphate 3-Kinase
PIK3CA	Phosphatidylinositol-4,5-Bisphosphate 3-Kinase Catalytic Subunit Alpha
QoL	Quality of Life
RT	Radiotherapy
SG	Sacituzumab Govitecan
SNOMR	National Society for Medical Oncology from
T-DXd	Trastuzumab Deruxtecan
TT	Targeted Therapy
